# Scoping Review: Home and Community-Based Service Waiver Programs and Person-Reported Outcomes

**DOI:** 10.1093/geroni/igab046.3082

**Published:** 2021-12-17

**Authors:** Taylor Bucy, John Mulcahy, Eric Jutkowitz, Tetyana Shippee

**Affiliations:** 1 University of Minnesota, University of Minnesota, Minnesota, United States; 2 Brown University, Brown University, Rhode Island, United States

## Abstract

State Medicaid programs are rebalancing their long-term care spending from nursing home to home and community-based services (HCBS). Emphasis on person-centered and person-directed care warrants investigation into models of HCBS delivery that promote quality of life. We performed a scoping review of the literature to catalogue the breadth of the studies describing HCBS waiver programs targeting adults (18+). We identified 757 articles, and after duplicate removal and reconciliation, we excluded articles on children or adolescents, non-peer reviewed reports, international studies, and articles that did not describe HCBS waiver programs. After abstract and title review, 292 articles met our inclusion criteria. Most included articles (22.3%) were single state descriptive evaluations or evaluations of service use patterns among participants. 17.8% of included articles examined multi-state or national variation in program trends, while 17.1% made national program conclusions without a major focus on interstate comparison. Less common were studies examining integrated care or dual-eligibles (7.5%), PACE (3.4%), medication management (3.1%), quality and satisfaction of both consumer and caretaker perspectives (3.8%) and consumer-only perspectives (5.1%). The remaining articles focused on HIV (4.1%), TBI (1.4%) or ID/DD (14.4%) waiver programs. The 8.9% of articles addressing quality and satisfaction consisted mostly of interviews, either with state Medicaid administrators or with care recipients and/or caregivers. Consumer reported satisfaction and unmet care needs were the primary outcomes examined. Given the heightened focus on long-term care as a result of the ongoing coronavirus pandemic, this review justifies further exploration into the delivery and outcomes of state-directed HCBS waiver programs.

